# Improvement and enhancement of oligosaccharide production from *Lactobacillus acidophilus* using statistical experimental designs and its inhibitory effect on colon cancer

**DOI:** 10.1186/s12934-023-02153-8

**Published:** 2023-08-09

**Authors:** Gaber A. Abo-Zaid, Ahmed M. Kenawy, Nehal M. El-Deeb, Lamiaa A. Al-Madboly

**Affiliations:** 1 Bioprocess Development Department, Genetic Engineering and Biotechnology Research Institute, City of Scientific Research and Technological Applications (SRTA-City), New Borg El-Arab City, Alexandria Egypt; 2 Nucleic Acids Research Department, Genetic Engineering and Biotechnology Research Institute, City of Scientific Research and Technological Applications (SRTA-City), New Borg El-Arab City, Alexandria Egypt; 3 Pharmaceutical Bioproducts Research Department, Genetic Engineering and Biotechnology Research Institute, City of Scientific Research and Technological Applications (SRTA-City), New Borg El-Arab City, Alexandria Egypt; 4 Department of Pharmaceutical Microbiology, Faculty of Pharmacy, Tanta University, Tanta, Egypt

**Keywords:** Lactic acid bacteria, Anticancer, Colon cancer, Production optimization

## Abstract

Colorectal cancer (CRC) is the third cause of death by cancers worldwide and is one of the most common cancer types reported in both Egypt and the United States. The use of probiotics as a dietary therapy is increasing either as a prevention or as a treatment for many diseases, particularly, in the case of CRC. The increasing acceptance of lactic acid bacterial (LAB) oligosaccharides as bioactive agents has led to an increase in the demand for the large-scale production of LAB-oligosaccharides using fermentation technology. Therefore, in the current study, we are using the Plackett- Burman design (PBD) approach, where sixteen experimental trials were applied to optimize the production of the target oligosaccharide LA-EPS-20079 from *Lactobacillus acidophilus*. Glucose, yeast extract and sodium acetate trihydrate were the top three significant variables influencing LA-EPS production. The maximum concentration of LA-EPS-20079 achieved by *L. acidophilus* was 526.79 μg/ml. Furthermore, Box–Behnken design (BBD) as response surface methodology (RSM) was used to complete the optimization procedure. The optimal levels of the chosen variables which were 30.0 g/l, glucose; 5 g/l, yeast extract and 10.0 g/l sodium acetate trihydrate with the predicted LA-EPS-20079 concentration of 794.82 μg/ml. Model validity reached 99.93% when the results were verified. Both optimized trials showed great cytotoxic effects against colon cancer line (CaCo-2) with inhibition percentages ranging from 64.6 to 81.9%. Moreover, downregulation in the expression level of *BCL*_*2*_ and *Survivin* genes was found with a fold change of 3.377 and 21.38, respectively. Finally, we concluded that the optimized LA-EPS-20079 has maintained its anticancer effect against the CaCo-2 cell line that was previously reported by our research group.

## Introduction

Cancer is one of the furthermost causes of health problems that lead to high mortality rates among patients. Worldwide, CRC comes in third place in the cancer death toll [1]. Surgery, chemotherapy, immunotherapy, and/or radiotherapy are the currently available approaches for treating patients suffering from cancer. Most of the chemotherapeutic drugs used for cancer treatment may cause immunotoxicity due to the lack of selectivity toward cancer cells. Furthermore, resistance to conventional chemotherapeutic agents might be developed by cancer cells [2]. Accordingly, there is a need for alternative therapies with high selectivity toward killing cancer cells or even using them to reduce the therapeutic doses and hence the side effects associated with highly toxic drugs.

Exo-poly/penta-saccharides (EPS) synthesized by lactic acid bacteria (LAB) are famous to have various biological activities. These natural compounds are usually synthesized during fermentation and then could be released in the fermentation medium [3]. Multiple health benefits were shown for exopolysaccharides extracted from LAB including antibacterial, anti-biofilm, and anti-tumor activities in addition to their immunomodulatory properties [4–6]. Many research groups have published data about these effects. For instance, Joo Seo et al. [7] stated that exopolysaccharides from *Lactobacillus plantarum* YML009 revealed a strong antioxidant effect. Also, exopolysaccharides obtained from *L. plantarum* 70,810 demonstrated anti-proliferative activities against Hepatocellular carcinoma [8].

EPSs are categorized into two groups; the first group is the homopolysaccharides group, which consists of a single type of monosaccharide unit, meanwhile, the second one is the heteropolysaccharides, which comprises a polymer chain consisting of two or more of different units of monosaccharides [6]. Instantaneously, the growth circumstances, which are used in cultivating the probiotic bacteria, might change the chemical composition and hence the structure of the exopolysaccharides [9]. Furthermore, experimental work has shown that the compositions of the exopolysaccharides and their structures are strain-dependent as reported by Li et al. [10]. Additionally, growth conditions might also affect the total amount of exopolysaccharides produced by LAB [9]. Specifically, the composition of production media is an effective component in maximizing the number of excreted exopolysaccharides. For example, Oleksy-Sobczak and Klewicka [11] reported that carbon sources, such as sucrose and fructose, influence the exopolysaccharide yields significantly.

Optimization of production media is an important step toward maximizing the production of the target product. Statistical experimental design became an easy approach to optimize the production media and help researchers to extract huge datasets from the minimum number of analyses. Plackett-Burman design (PBD) is one important approach of experimental designs that are usually used for screening a large number of variables that may affect the production of microbial target products [12, 13]. In addition, Response surface methodology (RSM) such as the Box-Behnken design is an important approach for analyzing the interaction between independent variables [14].

Several researchers used Plackett-Burman and Box-Behnken experimental designs in optimizing the production media for EPS production from *Lactobacillus* bacteria. For example, Chen et al. [15] used the Plackett-Burman design to optimize the medium for the cultivation of *L*. *acidophilus* and reported that sodium acetate trihydrate was an important variable in cultivating *L*. *acidophilus*. Also, Liu et al. [16] reported that sodium acetate trihydrate was the most significant variable influencing the yield of EPS from *L*. *acidophilus*. The model of the Box-Behnken design was sufficient in predicting the optimized values of all variables affecting *L*. *bulgaricus* [17].

In the current study, we conducted both Plackett-Burman and Box-Behnken designs to optimize the growth conditions and culture parameters for producing LA-EPS-20079 from *L. acidophilus* DSMZ 20079. Also, we evaluated its activity as an antitumor agent against human colon cancer cell line selectively and identified the mechanism of action at the molecular level.

## Materials and methods

### Cultivation of microorganisms

*L. acidophilus* DSMZ 20079 was purchased from DSMZ, this strain was selected based on the previously published data by our research group, which confirmed the presence of a positive anti-colon cancer effect of the *L*. *acidophilus* DSMZ 20079 bacterial Exo-Penta-Saccharide (LA-EPS) [3]. A separate colony of *L*. *acidophilus* DSMZ 20079 was inoculated into 50 ml of MRS medium in a 250-ml flask. Then, flasks were incubated overnight at 30 °C in a shaking incubator at 180 rpm.

### Extraction and quantitative assay of LA-EP- 20079

For the extraction of LA-EPS-20079, bacteria were harvested from the grown culture at the end of the incubation time using a 3,500 rpm centrifugation force for 30 min. Then, tri-chloro-acetic acid (TCA) was added to the culture supernatant and kept overnight at 4 °C. The following day, three cycles of ethanol precipitations were repeated and the LA-EP- 20079 was pelleted using centrifugation at 3,500 rpm for 30 min. Ethanol was evaporated in a rotary evaporator at 40 °C and the EPS was then dried in an oven at the same temperature [3].

Quantitative determination of LA-EPS-20079 was carried out using the phenol-sulfuric acid method [18] and glucose was used as a standard. Briefly, 200 μl of 5.0% phenol was added to the 200 μl of soluble LA-EPS-20079, followed by the addition of 1.0 ml of concentrated H_2_SO_4_. The reaction was boiled at 95 °C for 5 min, incubated on ice, and the absorbance was measured at 490 nm against blank samples.

### Statistical experimental designs

#### Plackett–Burman design

For determining the optimum combination of production factors including medium components and culture parameters, the Plackett–Burman factorial design was employed. Two levels were assigned for each variable: -1 for a low level and + 1 for a high level [12], the design was executed according to the model of the first order:

$$Y\, = \,{\beta _{\bf{0}}}\, + \,\sum \,\beta ixi$$

where ***Y*** represents the response (LA-EPS-20079 concentration), ***β***_**0**_ is the intercept, ***βi*** represents the linear coefficient, and ***xi*** is the degree of the independent variable.

Table 1 shows the design’s matrix, the tested factors and their levels used in the experimental design. As illustrated in Table 1, thirteen variables were screened using sixteen experiments. All experiments were carried out using three replicates and the mean of LA-EPS-20079 concentrations of the replicates was taken as the response value.

**Table 1:** Trials and variables levels in the Plackett–Burman experimental design and the resulted LA-EPS-20079 concentrations

Trial	pH	Incubation time (h)	Temperature (^o^C)	Glucose (g/l)	Peptone (g/l)	Beef extract (g/l)	Yeast extract (g/l)	K_2_HPO_4_ (g/l)	Triammonium citrate (g/l)	Sodium acetate trihydrate (g/l)	MgSO_4_.7H_2_O (g/l)	Tween-80 (ml/l)	Manganous sulfate tetrahydrate (g/l)	LA-EPS-20079 concentration (μg/ml)
1	+ 1 (6.5)	+ 1 (48)	+ 1 (37)	+ 1 (20)	+ 1 (10)	+ 1 (10)	+ 1 (5)	+ 1 (2)	-1 (0.2)	-1 (0.5)	-1 (0.2)	-1 (0.1)	-1 (0.05)	**417.02**
2	+ 1 (6.5)	+ 1 (48)	+ 1 (37)	+ 1 (20)	+ 1 (10)	+ 1 (10)	+ 1 (5)	-1 (0.2)	-1 (0.2)	-1 (0.5)	-1 (0.2)	-1 (0.1)	-1 (0.05)	**459.64**
3	+ 1 (6.5)	+ 1 (48)	+ 1 (37)	+ 1 (20)	+ 1 (10)	+ 1 (10)	-1 (0.5)	-1 (0.2)	-1 (0.2)	-1 (0.5)	-1 (0.2)	-1 (0.1)	-1 (0.05)	**376.79**
4	+ 1 (6.5)	+ 1 (48)	+ 1 (37)	+ 1 (20)	+ 1 (10)	-1 (1)	-1 (0.5)	-1 (0.2)	-1 (0.2)	-1 (0.5)	-1 (0.2)	-1 (0.1)	+ 1 (0.1)	**325.83**
5	+ 1 (6.5)	+ 1 (48)	+ 1 (37)	+ 1 (20)	-1 (1)	-1 (1)	-1 (0.5)	-1 (0.2)	-1 (0.2)	-1 (0.5)	-1 (0.2)	+ 1 (1)	+ 1 (0.1)	**183.21**
6	+ 1 (6.5)	+ 1 (48)	+ 1 (37)	-1 (2)	-1 (1)	-1 (1)	-1 (0.5)	-1 (0.2)	-1 (0.2)	-1 (0.5)	+ 1 (2)	+ 1 (1)	+ 1 (0.1)	**32.74**
7	+ 1 (6.5)	+ 1 (48)	-1 (30)	-1 (2)	-1 (1)	-1 (1)	-1 (0.5)	-1 (0.2)	-1 (0.2)	+ 1 (5)	+ 1 (2)	+ 1 (1)	+ 1 (0.1)	**128.45**
8	+ 1 (6.5)	-1 (24)	-1 (30)	-1 (2)	-1 (1)	-1 (1)	-1 (0.5)	-1 (0.2)	+ 1 (2)	+ 1 (5)	+ 1 (2)	+ 1 (1)	+ 1 (0.1)	**1.55**
9	-1 (6)	-1 (24)	-1 (30)	-1 (2)	-1 (1)	-1 (1)	-1 (0.5)	+ 1 (2)	+ 1 (2)	+ 1 (5)	+ 1 (2)	+ 1 (1)	+ 1 (0.1)	**3.45**
10	-1 (6)	-1 (24)	-1 (30)	-1 (2)	-1 (1)	-1 (1)	+ 1 (5)	+ 1 (2)	+ 1 (2)	+ 1 (5)	+ 1 (2)	+ 1 (1)	+ 1 (0.1)	**112.97**
11	-1 (6)	-1 (24)	-1 (30)	-1 (2)	-1 (1)	+ 1 (10)	+ 1 (5)	+ 1 (2)	+ 1 (2)	+ 1 (5)	+ 1 (2)	+ 1 (1)	+ 1 (0.1)	**137.27**
12	-1 (6)	-1 (24)	-1 (30)	-1 (2)	+ 1 (10)	+ 1 (10)	+ 1 (5)	+ 1 (2)	+ 1 (2)	+ 1 (5)	+ 1 (2)	+ 1 (1)	-1 (0.05)	**13.45**
13	-1 (6)	-1 (24)	-1 (30)	+ 1 (20)	+ 1 (10)	+ 1 (10)	+ 1 (5)	+ 1 (2)	+ 1 (2)	+ 1 (5)	+ 1 (2)	-1 (0.1)	-1 (0.05)	**419.88**
14	-1 (6)	-1 (24)	+ 1 (37)	+ 1 (20)	+ 1 (10)	+ 1 (10)	+ 1 (5)	+ 1 (2)	+ 1 (2)	+ 1 (5)	-1 (0.2)	-1 (0.1)	-1 (0.05)	**399.17**
15	-1 (6)	+ 1 (48)	+ 1 (37)	+ 1 (20)	+ 1 (10)	+ 1 (10)	+ 1 (5)	+ 1 (2)	+ 1 (2)	-1 (0.5)	-1 (0.2)	-1 (0.1)	-1 (0.05)	**379.17**
16	-1 (6)	-1 (24)	-1 (30)	-1 (2)	-1 (1)	-1 (1)	-1 (0.5)	-1 (0.2)	-1 (0.2)	-1 (0.5)	-1 (0.2)	-1 (0.1)	-1 (0.05)	**239.41**

The values of LA-EPS-20079 production were analyzed using multiple linear regressions to estimate multiple correlation coefficient R, determination of coefficient (R^2^), *t*-values, *P*-values and confidence levels, expressing the *P*-values as a percentage. In order to confirm the results, four conformational trials were carried out to confirm the previously obtained results of the Plackett–Burman design (Table 2).

**Table 2:** Confirmation trials of Plackett–Burman design

Variable	Trial 1	Trial 2	Trial 3	Trial 4
PH	6	6	6	6
Incubation time (h)	48	48	48	48
Temperature (ºC)	30	30	30	30
Glucose (g/l)	20	20	20	20
Peptone (g/l)	1	-------	1	1
Beef extract (g/l)	10	10	10	10
Yeast extract (g/l)	5	5	5	5
K_2_HPO_4_ (g/l)	0.2	-------	0.2	0.2
Triammonium citrate (g/l)	0.2	-------	0.2	0.2
Sodium acetate trihydrate (g/l)	5	5	5	5
MgSO_4_.7H_2_O (g/l)	2	2	2	2
Tween-80 (ml/l)	0.1	-------	-------	-------
Manganous sulfate tetrahydrate (g/l)	0.1	0.1	0.1	-------

#### Response surface methodology (Box-Behnken design)

LA-EPS-20079 production optimization using response surface methodology was applied using Box-Behnken design (BBD) to optimize the tested variables [19].

After assessing the significance of the variables using the PBD, the highest three variables regarding their significant effect on the LA-EPS-20079 production (glucose, yeast extract, and sodium acetate trihydrate) resulted from Plackett-Burman design were chosen to define their optimal levels for LA-EPS-20079 production using BBD. A software (JMP) was used to analyze the obtained data set at three levels: low, medium, and high represented by -1, 0, and + 1, respectively as presented in Table 3. Three steps of optimization were carried out as follows: statistical design, determining the coefficients of the mathematical model, and model adequacy assessment and response prediction. Table 3 shows the matrix of the experimental design.

**Table 3:** Box–Behnken factorial design demonstrating LA-EPS-20079 concentration as a response to changing glucose, yeast extract, and sodium acetate trihydrate levels

Trial	Glucose (g/l)	Yeast extract (g/l)	Sodium acetate trihydrate (g/l)		Oligosaccharides concentration (μg/ml)	
					Measured	Predicted
1	-1 (20)	-1 (5)	0 (7.5)	485		480.65
2	+ 1 (30)	-1 (5)	0 (7.5)	543.095		583.75
3	-1 (20)	+ 1 (10)	0 (7.5)	442.62		401.96
4	+ 1 (30)	+ 1 (10)	0 (7.5)	675		679.3
5	-1 (20)	0 (7.5)	-1 (5)	423.57		454.70
6	+ 1 (30)	0 (7.5)	-1 (5)	516.90		503.045
7	-1 (20)	0 (7.5)	+ 1 (10)	417.86		431.73
8	+ 1 (30)	0 (7.5)	+ 1 (10)	794		763.87
9	0 (25)	-1 (5)	-1 (5)	458.33		431.55
10	0 (25)	+ 1 (10)	-1 (5)	571.19		580.714
11	0 (25)	-1 (5)	+ 1 (10)	700.71		691.19
12	0 (25)	+ 1 (10)	+ 1 (10)	532.14		558.93
13	0 (25)	0 (7.5)	0 (7.5)	545.95		546.90
14	0 (25)	0 (7.5)	0 (7.5)	547.86		546.90

Regression analysis was performed using Microsoft Excel and JMP software. Coefficient of determination (R^2^) was calculated to determine the model’s quality of fit. For the three tested variables, we applied the following second-order polynomial model:

$$\begin{array}{l}Y = {b_0}_{} + {b_1}{X_1} + {b_2}{X_2} + {b_3}{X_3} + {b_{12}}{X_1}{X_2} + {b_{13}}{X_1}{X_3}\\+ {b_{23}}{X_2}{X_3} + {b_{11}}{X_1}^2 + {b_{22}}{X_2}^2 + {b_{33}}{X_3}^2\end{array}$$

*Y*: the predicted response of LA-EPS-20079 concentration; *β*_*0*_: model intercept; *X*_*1*_, *X*_*2*,_ and *X*_*3*_ are the independent variables, *β*_*1*_, *β*_*2*_ and *β*_*3*_ are linear coefficients; *β*_*12*_, *β*_*13*_ and *β*_*23*_ are cross-product coefficients; and *β*_*11*_, *β*_*22*_ and *β*_*33*_ are the quadratic coefficients [19–21].

A conformational trial was carried out to confirm the previously obtained results of the Box- Behnken design.

Verification of the model was carried out using triplicate trials. The predicted value of LA-EPS-20079 oligosaccharides concentration resulting from the model was compared to the measured value of LA-EPS-20079 concentration. The accuracy percentage was calculated using the following equation:

$${Y_{accuracy}}\% \, = \,\left( {Y\,exp/Y\,calc} \right)\,*100$$

#### Statistical analysis of data

Multiple linear regression analysis was performed using Microsoft Excel and JMP software to determine the *t*-values, *P*-values, and confidence levels expressing the *P*-values as a percentage. The predicted values of LA-EPS-20079 concentration and the optimal values of the most significant variables were determined by JMP software. The three-dimensional graphs were generated using Statistica 7.0 software to represent the simultaneous effects of the tested variables on the LA-EPS-20079 concentration.

### Purification of the active compound from LA-EPS-20079

The optimized crude LA-EPS-20079 sample was purified using HPLC (Agilent, USA) with C18 Column (Biorad) and refractive index detector using a flow rate of 0.8 ml/min to detect the most potent compound in the semi-purified fraction of LA-EPS which has anticancer activity.

### Evaluation of the anti-cancer effect of the extracted oligosaccharide

The anticancer effect of the optimized LA-EPS-20079 against the CaCo-2 cell line was investigated using an MTT assay. About 100 μl CaCo-2 cell suspension (6 × 10^4^ cell/ml) was distributed in the wells of 96-well tissue culture plates. The plates were then incubated at 37^o^C in a humidified CO_2_ incubator (5.0%) until semi-confluent cells grew in the plates. Then, the old medium was discarded and exchanged with 100 μl of RPMI medium containing 5 mg/ml LA-EPS-20079 obtained from the trials of Box-Benkhen design, and their verification experiments. Also, to set up an experimental negative control, 100 μl of the used medium was then added. All plates were finally incubated as described above for 24 h and an MTT assay was used to assess cellular viability. Also, IC50 value of the test LA-EPS-20079 was determined [22].

### Induction of apoptosis and assessment of necrosis

To distinguish between both apoptosis as well as necrosis cellular status, the assay of acridine orange and ethidium bromide (AO/EB) was followed as described by Kasibhatla et al. [23]. For acridine orange, this dye could stain both live as well as dead cells however; ethidium bromide dye stains only cells with damaged membrane integrity. Following treatment with LA-EPS-20079 oligosaccharides, cell suspension of CaCo-2 (0.5 × 10^6^ cells/ml) was incubated with about 1 μl of the staining AO/EB solution. Examination of 10 μl of cell suspension, applied to a microscopic slide, was caried out using a fluorescence microscope.

### Effect of LA-EPS-20079 on gene expression

Transcription abundance of both *BCL2* and *Survivin* genes were analyzed in both LA-EPS-20079 treated and untreated (control) CaCo-2 cell line. Two-steps RT-qPCR technique was applied and *GAPDH* gene was used as an internal reference gene for gene expression data normalization. RNA extraction was done using a TRIzol-based RNA extraction kit according to the manufacturer protocol. Then, cDNA synthesis was carried out using a cDNA synthesis kit (Thermo Scientific) using 10 μl of the obtained RNA (as per the manufacturer’s instructions).

qPCR reactions were performed in triplicates, where 1 μl of the cDNA for each sample was incorporated with Maxima SYBR Green Master Mix and 5 pmol of each forward and reverse gene-specific primers (*BCL2*-F: 5’-TATAAGCTGTCGCAGAGGGGCTA3’, BCL2-R: 5’-GTACTCAGTCATCCACAGGGCGAT3’, *Survivin*-F: 5’-TGCCCCGACGTTGCC-3, -R: 5’-CAGTTCTTGAATGTAGAGATGCGGT-3’). In addition, *GAPDH* gene-specific primers (*GAPDH*-F: 5’-GAA GGT GAA GGT CGG AGT-3 and *GAPDH*-R: 5’-GAA GAT GGT GAT GGG ATT TC-3’) were used for determining the internal reference gene expression level. The PCR cycle program was done in a BioRad Real-Time PCR (BioRad, USA) using the following cycling program: 1cycle 95 °C for 1 min then 40 cycles of; 15 s at 94 °C, 30 s at 50 °C, and 30 s at 72 °C. The Cq values were normalized then the mean relative gene expression levels and fold expression were analyzed by CFX manager Software version 3.0.1 (BioRad, USA) [24].

## Results and discussion

### Plackett–Burman design

#### Evaluation of factors affecting LA-EPS-20079 production

**Fig. 1:**
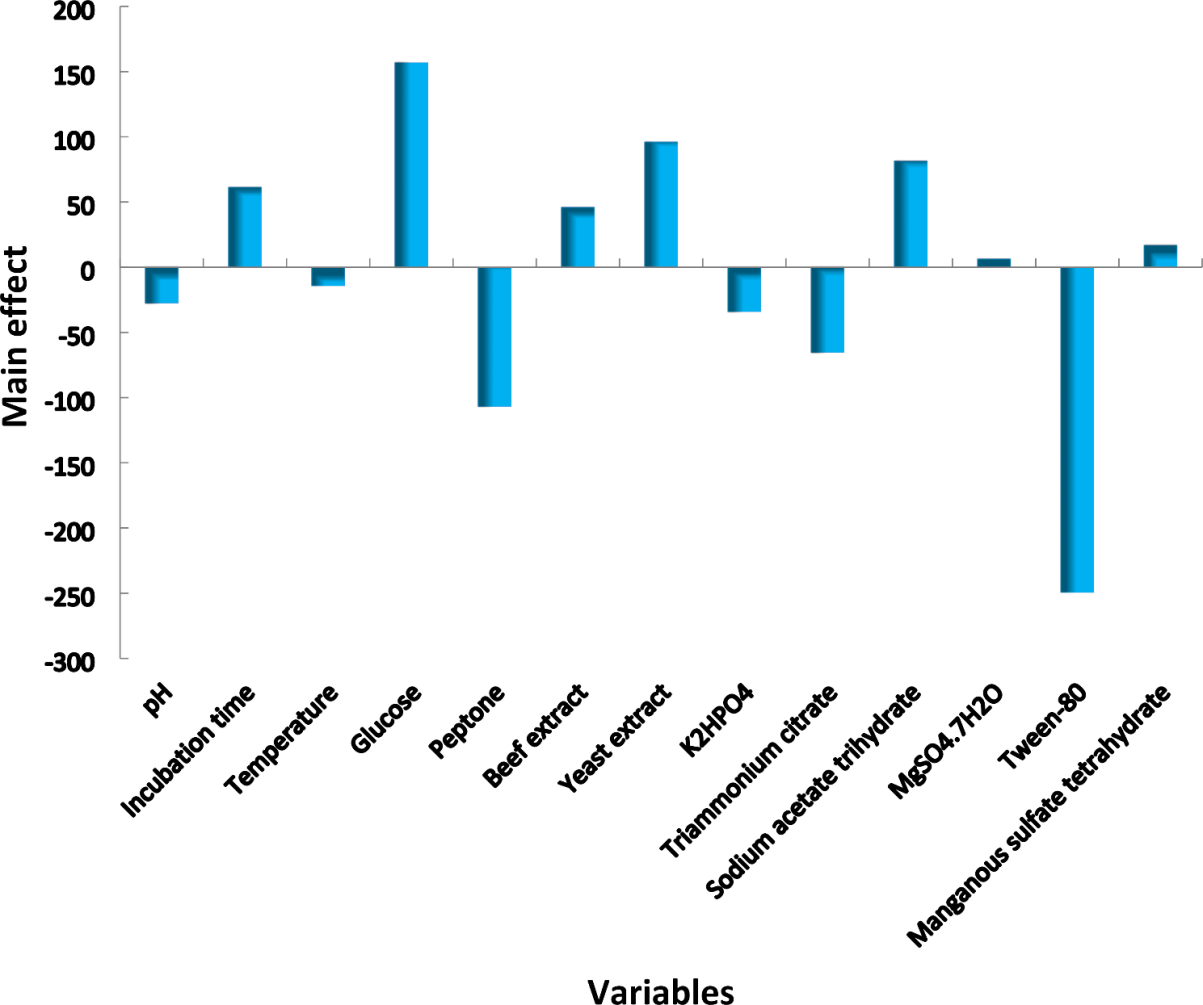
Effect of optimization factors on LA-EPS-20079 oligosaccharide concentrations (μg/ml) produced by *Lactobacillus acidophilus*

**Fig. 2:**
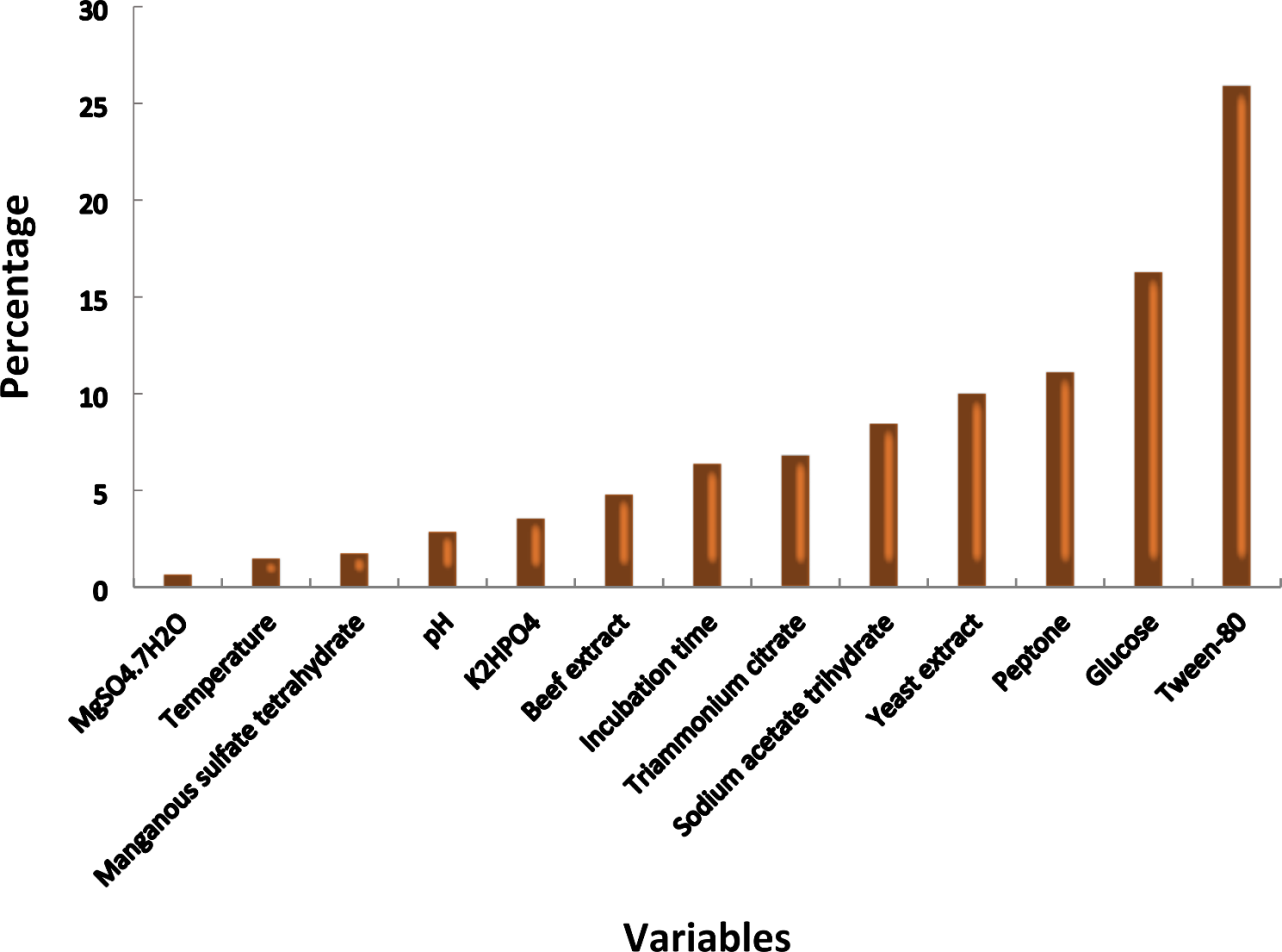
Pareto chart rationalizing the effect of each variable on LA-EPS-20079 concentration (μg/ml) produced by *Lactobacillus acidophilus*

Plackett–Burman design was used to determine the importance of the tested thirteen different factors (variables) and media conditions (Table 1) and to optimize LA-EPS production. The results presented in Table 1 show the mean values of LA-EPS-20079 concentrations obtained from different experiments (given in μg/ml), where variability that ranged between 1.55 and 459.64 μg/ml of LA-EPS-20079 was observed. In addition, the main effect was calculated for each variable and presented graphically in Fig. 1. Regression coefficients analysis of the thirteen variables, including incubation time, glucose, yeast extract, beef extract, sodium acetate trihydrate, MgSO_4_.7H_2_O and MnSO_4_.4H_2_O indicated the presence of positive effects on LA-EPS-20079 production. On the other hand, pH, temperature, peptone, K_2_HPO_4_, triammonium citrate and tween 80 showed a negative effect on the oligosaccharide LA-EPS-20079 production. The data illustrated in Fig. 2 showed the ranking of the factors as a Pareto chart. Furthermore, the correlation between the tested variables and the response is presented by the polynomial model:

$$\begin{array}{*{20}{l}}{{{\bf{Y}}_{{\bf{activity}}}} = {\bf{226}}.{\bf{875}} - {\bf{13}}.{\bf{810}}{X_{\bf{1}}} + {\bf{30}}.{\bf{714}}{X_{\bf{2}}}}\\{ - {\bf{7}}.{\bf{143}}{X_{\bf{3}}} + {\bf{78}}.{\bf{452}}{X_{\bf{4}}} - {\bf{53}}.{\bf{452}}{X_{\bf{5}}} + {\bf{23}}.{\bf{036}}{X_{\bf{6}}}}\\{ + {\bf{48}}.{\bf{095}}{X_{\bf{7}}} - {\bf{17}}.{\bf{083}}{X_{\bf{8}}} - {\bf{32}}.{\bf{738}}{X_{\bf{9}}} + {\bf{40}}.{\bf{714}}{X_{{\bf{10}}}}}\\{ + {\bf{3}}.{\bf{214}}{X_{{\bf{11}}}} - {\bf{124}}.{\bf{762}}{X_{{\bf{12}}}} + {\bf{8}}.{\bf{452}}{X_{{\bf{13}}}}}\end{array}$$

The results obtained from the ANOVA analysis are summarized in Table 4. The obtained results indicated that the *p*-value of the model was 0.013 indicating a significant relationship. The determination coefficient (*R*^*2*^) indicated that 0.998 variabilities in LA-EPS-20079 concentration is explained by the model as fitted. The multiple *R* (correlation coefficient) indicated that there is a strong correlation of 0.991 between the measured and the predicted values of LA-EPS-20079 concentration.

**Table 4:** ANOVA analysis of the obtained data of Plackett-Burman design

Analysis of Variance						
Source	Degree of freedom	Sum of squares	Mean square	F-ratio	*P*-value	
Regression	13	428550.5	32965.42	76.34998	0.012999	
Residual	2	863.5346	431.7673			
Total	15	429,414	32965.42			

Correlation coefficient (Multiple *R*) = 0.998994

Determination coefficient (*R*-squared) = 0.997989

The data presented in Table 5 showed that glucose, yeast extract, and sodium acetate trihydrate (confidence level > 90%) were variable with the most significant effect on LA-EPS-20079 production. The remaining variables were omitted in the following optimization trials, but instead, they were kept at the either (-1) level or (+ 1) level, each according to their contribution. Gamar et al. [25] reported that the carbon source concentration was the main factor affecting production of EPS from *L. rhamnosus*. The obtained results in the current study were similar to these results reported by Imran et al. [26] who documented that EPS production medium containing 20 g/l of glucose gave the highest EPS yield from *L*. *plantarum* NTMI05 and NTMI20. Additionally, *Lactobacillus* spp. and *Lactobacillus sakei* produced the maximum concentration of EPS on glucose as a carbon source [27–29]. The Plackett-Burman design was utilized by Polak-Berecka et al. [24] for optimizing EPS production using *L*. *rhamnosus* strain E/N, which revealed a yeast extract was the main and important variable for EPS production. Biomass and EPS concentration of *L*. *rhamnosus* increased by adding yeast extract to the production medium [28]. Liu et al. [16] reported that sodium acetate trihydrate was the most significant variable influencing the yield of EPS from *L*. *acidophilus*.

**Table 5:** Statistics of Plackett–Burman data analysis indicating the coefficient, *t*-values, and *P*-values for each variable of the design

Variable	Coefficients	*t*-value	*P*-value	Confidence level (%)
Intercept	226.875	43.67388	0.000524	
pH	-13.8095	-1.02958	0.411432	58.86
Incubation time	30.71429	2.289925	0.149177	85.08
Temperature	-7.14286	-0.53254	0.647594	35.24
Glucose	78.45238	5.849071	0.028008	97.20
Peptone	-53.4524	-3.98518	0.057581	94.24
Beef extract	23.03571	1.862829	0.203521	79.65
Yeast extract	48.09524	4.629213	0.043633	95.64
K_2_HPO_4_	-17.0833	-1.38148	0.301221	69.88
Triammonium citrate	-32.7381	-2.44081	0.134745	86.53
Sodium acetate trihydrate	40.71429	3.035481	0.093549	90.65
MgSO_4_.7H_2_O	3.214286	0.239643	0.832928	16.71
Tween-80	-124.762	-9.30171	0.011361	98.86
Manganous sulfate tetrahydrate	8.452381	0.630173	0.592981	40.70

#### Confirmation of plackett–burman design

**Fig. 3:**
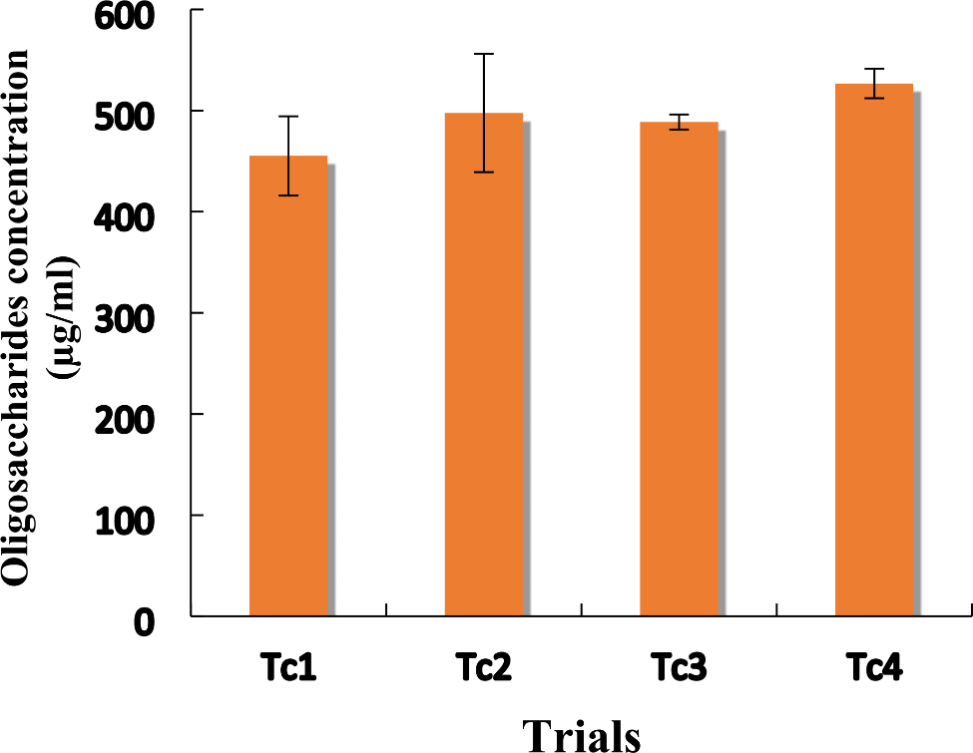
Confirmation trials of Plackett–Burman design representing the concentration of LA-EPS-20079 produced in each trial

The conformation experiment indicated that trial No. 4 (Tc4) gave a higher concentration of LA-EPS-20079 oligosaccharide than the other trials (Tc1, Tc2 and Tc3). Trial No. 4 (Tc4) achieved a concentration of LA-EPS-20079 equal to 526.79 μg/ml, meanwhile, Tc1, Tc2, and Tc3 trials resulted in concentrations of 455.20, 497.59, and 488.61 μg/ml, respectively, as illustrated in Fig. 3.

### Response surface methodology (Box-Behnken design)

Based on the Plackett-Burman design, the three most-significant variables inducing LA-EPS-20079 oligosaccharides production were determined to be glucose, X1; yeast extract, X2; and sodium acetate trihydrate, X3. These variables were investigated at three levels using fourteen different experimental trials to complete the response surface methodology (RSM), Box-Behnken design (BBD). Amiri et al. [30] used the Box-Behnken design (BBD) to optimize the production of LA-EPS from *L*. *acidophilus* LA5. In the present study, data presented in Table 3 showed the trials design for the tested variables along with the obtained results of LA-EPS-20079 concentration. Linear multiple regression analysis showed that the confidence levels of the X1 (glucose) and X3 (sodium acetate trihydrate) variables were 99.68 and 98.31%, respectively (Table 6). This indicates that they made more extensive contributions than the other medium constituent variables did.

**Table 6:** Box-Behnken statistics showing the coefficient values, *t*-values, and *P*-values for each variable of the design

Variable	Coefficients	*t*-statistics	*P*-value	Confidence level (%)
Intercept	546.9047619			
x1	95.11904762	6.304373	0.003236	99.67638
x2	4.226190476	0.280107	0.793285	20.67154
x3	59.46428571	3.941219	0.016942	98.30576
x1 × 2	43.57142857	2.042026	0.110688	88.93115
x1 × 3	70.95238095	3.325266	0.029234	97.07655
x2 × 3	-70.35714286	-3.29737	0.030007	96.99927
x1 × 1	-18.86904762	-0.79096	0.473224	52.67762
x2 × 2	8.392857143	0.351815	0.742729	25.72712
x3 × 3	10.29761905	0.431659	0.688237	31.17627

The results of the ANOVA analysis were shown in Table 7 as *P* = 0.024 indicating a significant relationship among the tested variables (*P* = 0.05). Correlation analysis of the tested model was done by calculating the determination coefficients (R^2^) and multiple correlation coefficients (R). The results indicated that R = 0.98 for LA-EPS-20079 oligosaccharides concentration, indicating a strong correlation between the measured and the predicted values. Also, the value of R^2^ = 0.95 indicated that only 5.0% of the total differences are not explained by the model. For predicting the optimal combination for optimizing the production of the LA-EPS, a second-order polynomial function was fitted to the experimental data using a linear optimization algorithm and the generated model combining the three variables is presented in the following equation:

**Table 7:** ANOVA analysis of the obtained data from Box-Behnken experimental design work

Analysis of variance					
Source	Degree of freedom	Sum of squares	Mean square	F-ratio	*P*-value
Regression	9	150473.4	16719.26	9.180704	0.023663
Residual	4	7284.524	1821.131		
Total	13	157757.9			

Correlation coefficient (Multiple *R*) = 0.98

Determination coefficient (*R*-squared) = 0.95

$$\begin{array}{l}Y = {\rm{ }}{\bf{546}}.{\bf{90}}{\rm{ }} + {\rm{ }}{\bf{95}}.{\bf{12}}{X_1} + {\rm{ }}{\bf{4}}.{\bf{23}}{X_2} + {\rm{ }}{\bf{59}}.{\bf{46}}{X_3}\\+ {\rm{ }}{\bf{43}}.{\bf{57}}{X_1}{X_2} + {\rm{ }}{\bf{70}}.{\bf{95}}{X_1}{X_3}-{\rm{ }}{\bf{70}}.{\bf{36}}{X_2}{X_3}\\-{\rm{ }}{\bf{18}}.{\bf{87}}{X_1}^{\bf{2}} + {\rm{ }}{\bf{8}}.{\bf{39}}{X_2}^{\bf{2}} + {\rm{ }}{\bf{10}}.{\bf{30}}{X_3}^{\bf{2}}\end{array}$$

Where *X*1, *X*2, and *X*3 are glucose, yeast extract, and sodium acetate trihydrate, respectively, and Y is the response (LA-EPS-20079 concentration).

#### Examination of the model’s adequacy

**Fig. 4:**
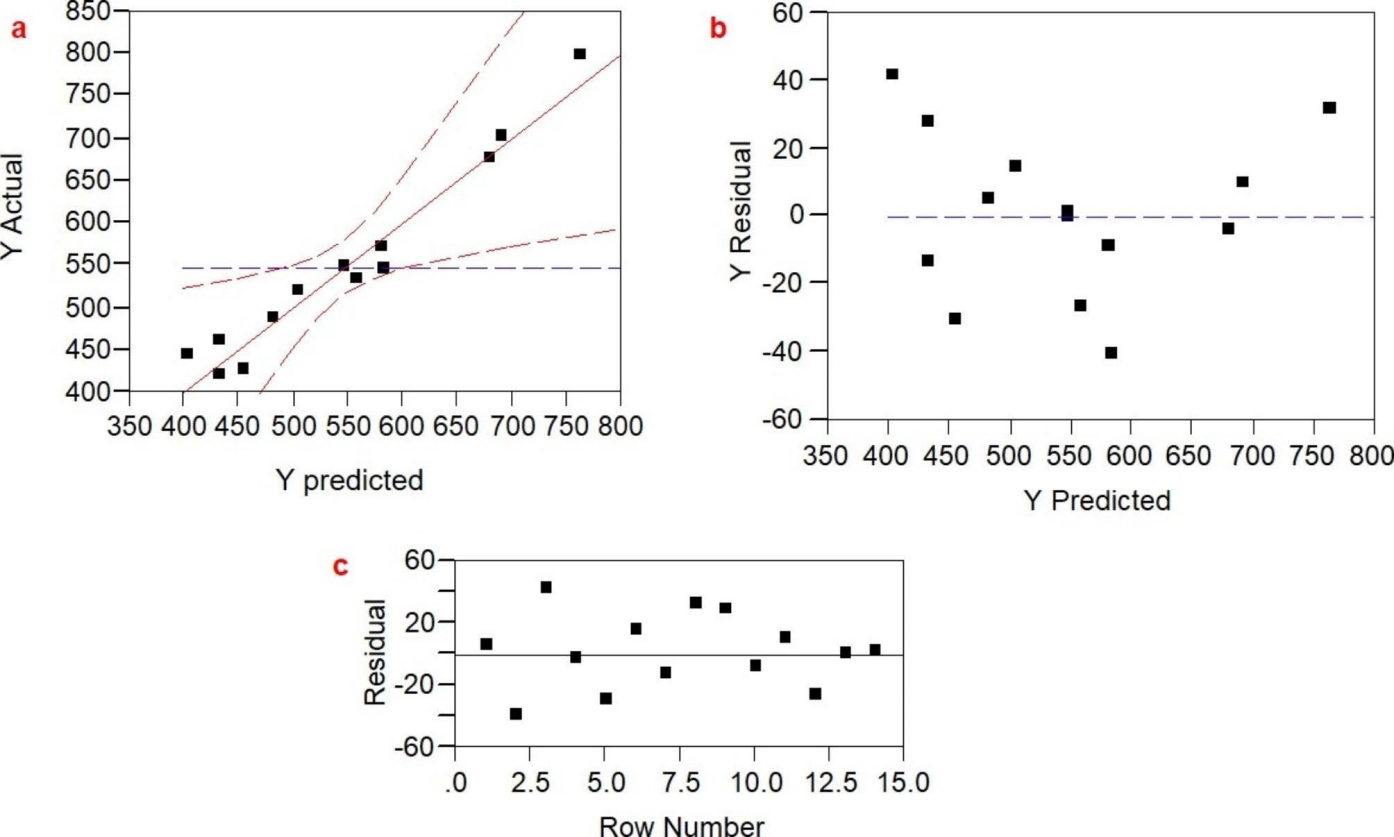
Model adequacy examination of Box-Behnken design (**a**) plot of the actual values versus the predicted values of LA-EPS-20079 oligosaccharides production, (**b**) the residuals versus the predicted LA-EPS-20079 oligosaccharides production and (**c**) the correlation between the residual and observation order

The data presented in Fig. 4a showed the distribution of experimental points and the predicted points around the central line, which appears to be regularly distributed, representing a normal distribution and the adequacy of the model. As shown in Fig. 4b, the residuals were plotted against their predicted response, which confirmed the postulation of constant variance. The random scatter plot indicated that the variance of the original observation was constant among LA-EPS-20079 concentrations. Therefore, data transformation was not needed for the response variables (within a 60 to -60 range). In addition, Fig. 4c illustrated that all data points were randomly distributed around the central line. This random distribution guarantees that the generated results are not biased.

**Fig. 5:**
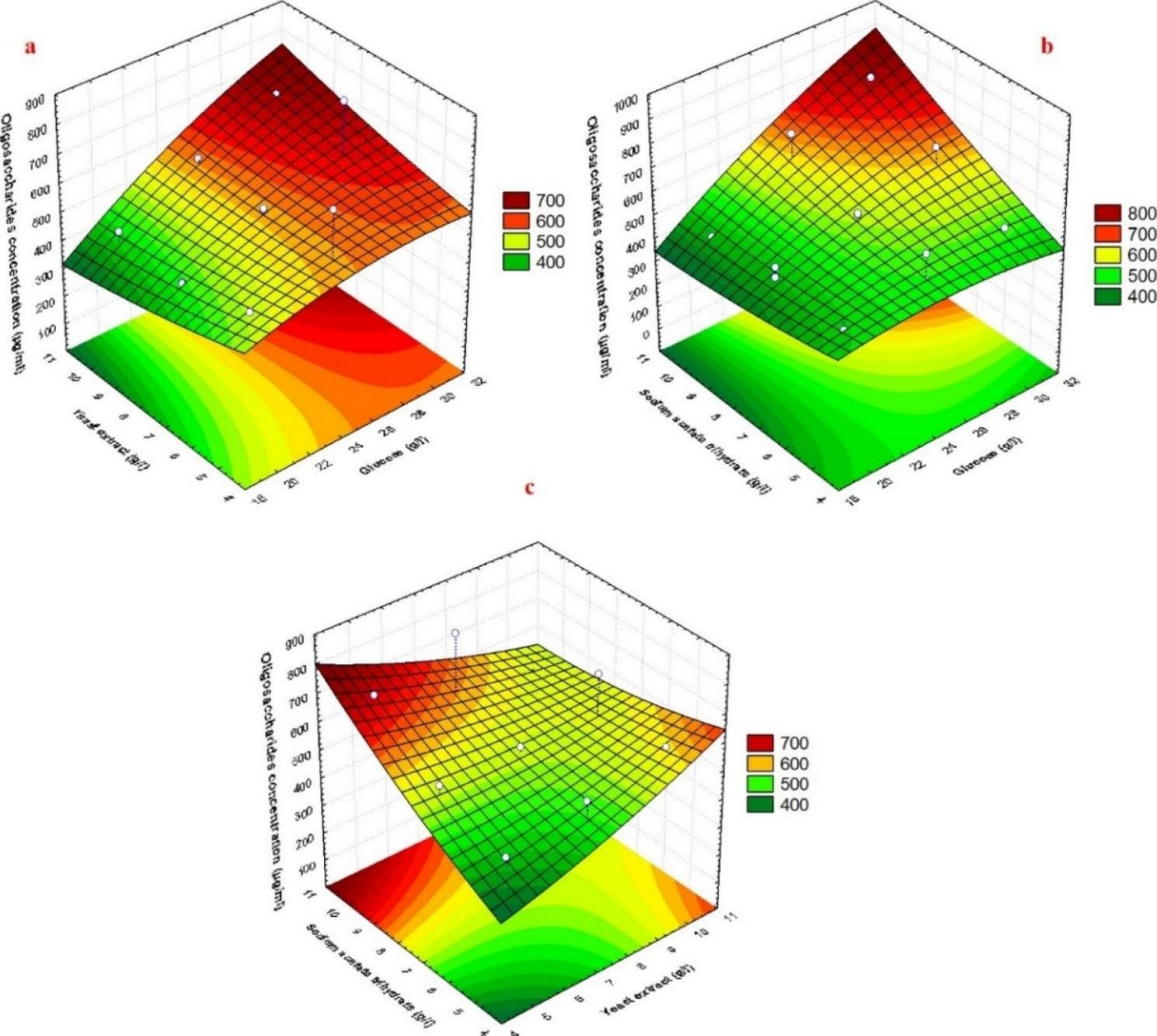
Three-dimensional response surface and contour plots representing LA-EPS-20079 oligosaccharides production by *Lactobacillus acidophilus* in terms of concentration (μg/ml) as affected by culture conditions (glucose, yeast extract, and sodium acetate trihydrate)

The generated 3D and contour plots were generated to figure out the interaction between the variables affecting LA-EPS-20079 oligosaccharides production. These plots showed that increasing glucose and sodium acetate trihydrate concentrations achieved higher levels of LA-EPS-20079 oligosaccharides concentration. Meanwhile, the lower levels of yeast extract supported a high level of oligosaccharides concentration (Fig. 5). Amiri et al. [30] reported that a high concentration of yeast extract resulted in a reduction in the amount of EPS produced from *L*. *acidophilus* LA5. This result is in settlement with the results obtained in the current study. Also, Macedo et al. [28] showed that EPS production by *L*. *rhamnosus* was improved by adding yeast extract in low concentration to the production medium.

**Fig. 6:**
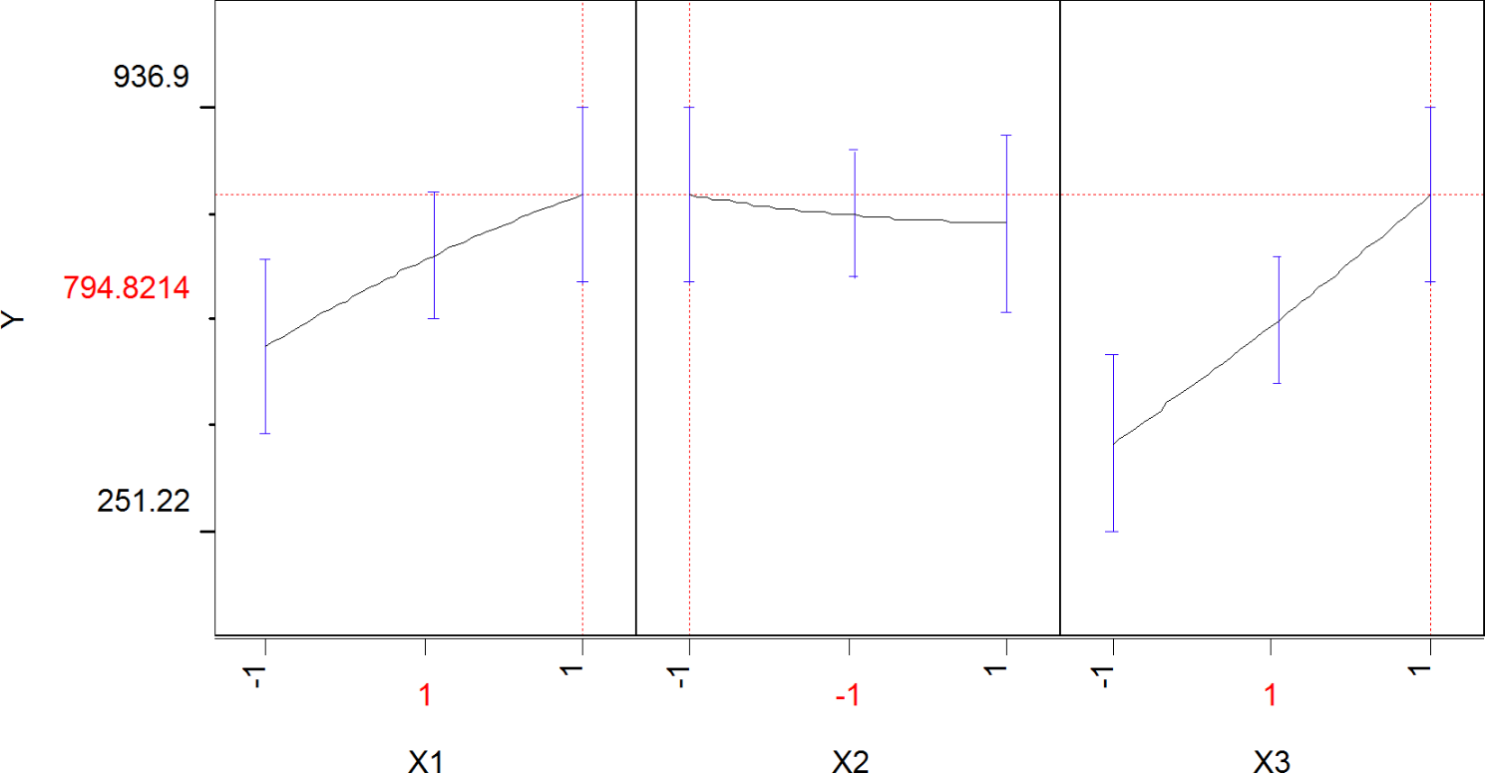
JMP profile showing the predicted optimal levels of the most significant variables, X1, glucose; X2, yeast extract and X3, sodium acetate trihydrate alongside the predicted LA-EPS-20079 oligosaccharides

Furthermore, the best levels of the three variables indicated by the maximum level of the polynomial model were determined by the solver function of the Microsoft Excel and JMP software. These points were as follows; 30 g/l, glucose; 5 g/l, yeast extract and 10 g/l, sodium acetate trihydrate with the predicted LA-EPS-20079 oligosaccharides concentration equal to 794.82 μg/ml (Fig. 6). Therefore, the obtained results of Plackett-Burman and Box-Behnken designs revealed that the expected medium constituents for optimum production of LA-EPS-20079 oligosaccharides by *L*. *acidophilus* is (in g/l): 30, glucose; 1.0, peptone; 10.0, beef extract; 5.0, yeast extract; 0.2, K_2_HPO_4_; 0.2, triammonium citrate; 10.0, sodium acetate trihydrate and 2.0, MgSO_4_.7H_2_O and physical variables; temperature 30 °C, pH 6, and 48 h incubation time.

#### Verification of the model

A verification experiment was done in order to assess the accuracy of the quadratic polynomial using the previously predicted optimal conditions. LA-EPS-20079 oligosaccharides concentration produced by *L*. *acidophilus* was 794.31 μg/ml. This result reflects a high level of accuracy of the model (99.93%) and assures the model’s validity under the investigated conditions.

#### Anticancer effects of the selected trial and purified fraction

**Fig. 7:**
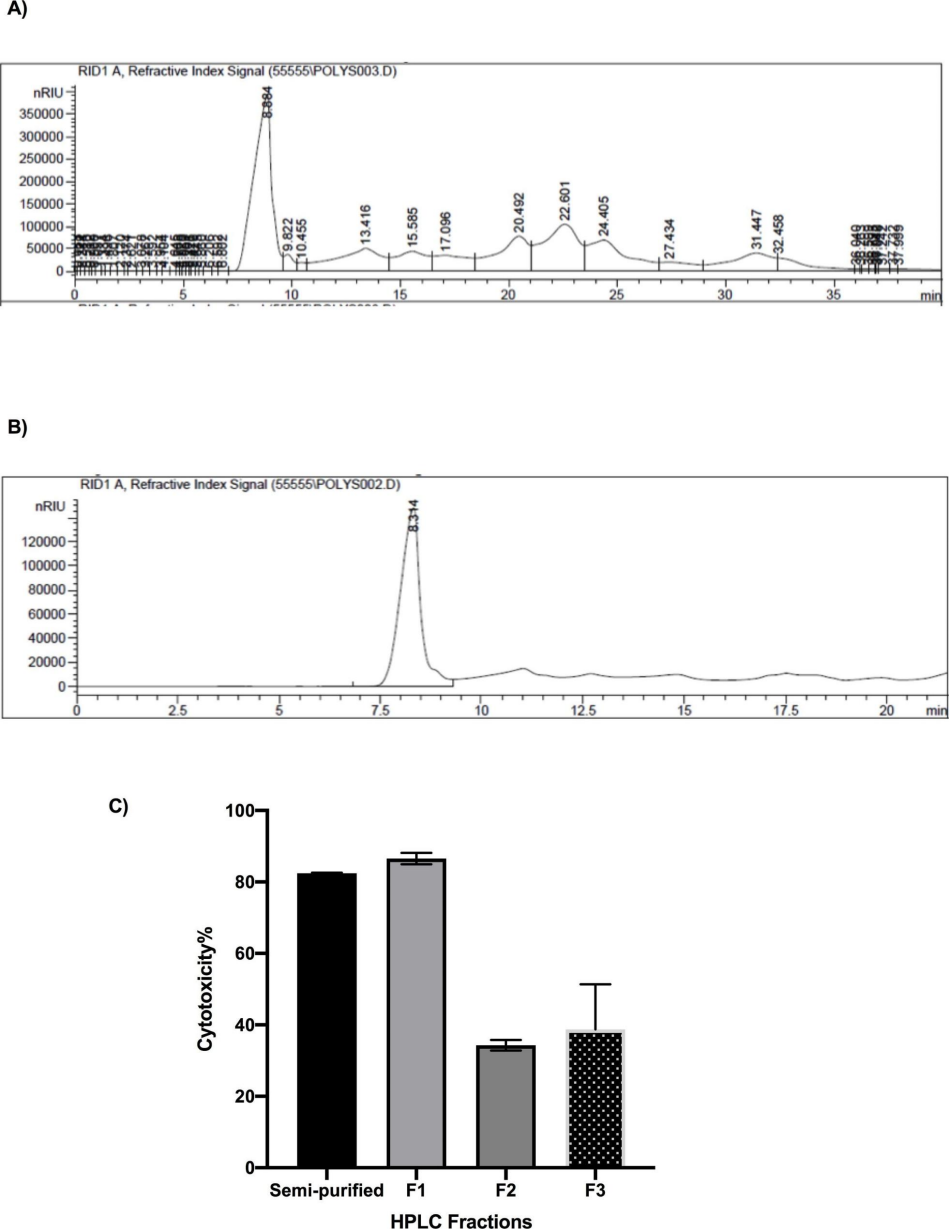
HPLC chromatograms of the semi-purified (**A**) and the purified (**B**) LA-EPS-20079 fractions (F1-F3) and their anticancer effects (**C**) tested against CaCo-2 cell line comparing to the semi-purified fraction

**Fig. 8:**
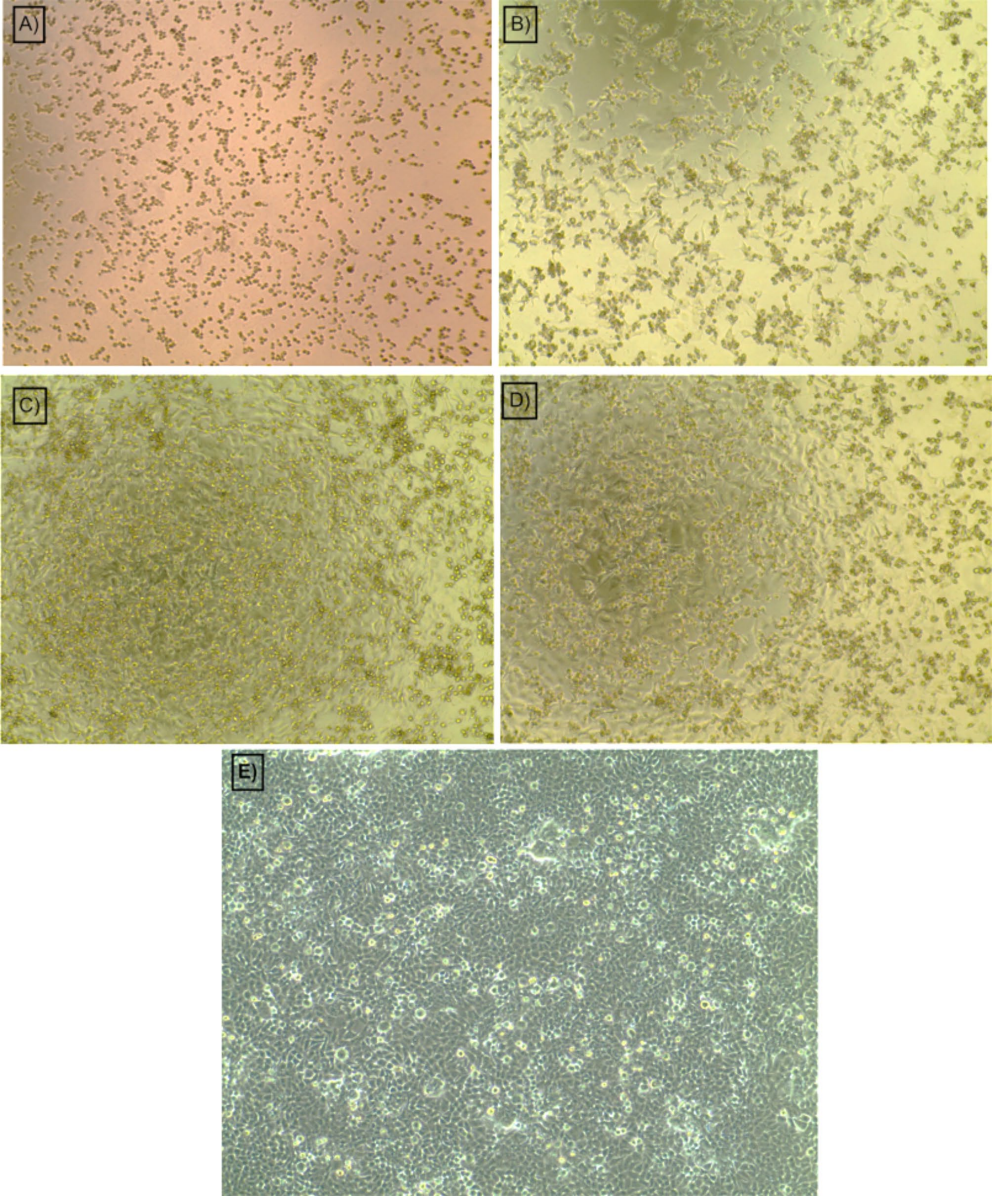
The morphological changes of CaCo-2 cell lines after treatment with the collected trails of Box-Behnken confirmation designs using 5 mg/ml of each trail

LA-EPS-20079 was further purified using HPLC and the extracted fraction was separated on C18 Column. The chromatogram presented in (Fig. 7A) indicated that the semi-purified fraction contains minor peaks as impurities. In order to clean out the associated compounds we run multiple HPLC runs and collected the selected compound that appears at RT 8.32 min. (Fig. 7b). The collected fractions were then dried out and the obtained compound was dissolved in 1 ml of purified water and its purity was assessed under the same HPLC conditions. In addition, the anticancer effect of the collected fraction showed high anticancer activity against CaCo-2 reaching 86.542% (Fig. 7C) compared with the activity of the semi-purified trail that showed an inhibition percentage of 82.2% (Fig. 7C). Furthermore, the concentration of the purified pentasaccharide the inhibited 50% of the cancer cells (IC50) was found to be 1.5 mg/ml. The morphological changes of the collected four trials of Box-Behnken confirmation designs on CaCo-2 cell lines presented in Fig. 8: trial 1 (Fig. 8A), trial 2 (Fig. 8B), trial 3 (Fig. 8C), trial 4 (Fig. 8D), and the untreated cells (Fig. 8E). The results indicated the occurrence of dead cells as well as the apoptotic effect of the treatment that existed in the majority of the inspected plates. Also, serious changes in the structure and number of the treated cells were observed. The anticancer activities of microbial EPS have been thoroughly identified and supported by research. For instance, the potent anticancer effect and the safety usage pattern of the purified pentasaccharide against two cell lines (CaCo-2 and MCF7 cell lines) were previously evaluated by our group. Additionally, El-Deeb et al. [3] reported thay a cytotoxicity, BrdU incorporation and annexin V quantification assays were carried out. Our previous study indicated that the cellular viability inhibition percentage imposed on CaCo-2-treated cells reached 80.65, which is slightly lower than the obtained result in the current study, which is represented by the optimization trial (82.0%). This result confirms the composition similarities between the originally extracted fraction and the newly optimized one. Similar results were recorded by Deepak et al. [31], they reported anticancer activities of EPS produced from probiotic *L. acidophilus* employing Plackett–Burman design and Central Composite Rotatory Design (CCRD) against CaCo-2 cell line. They found an inverse relationship between the EPS concentration and CaCo-2 cells survival. Also, they found that when 5 mg/ml was used, a significant depression in cell survival rate was found.

**Fig. 9:**
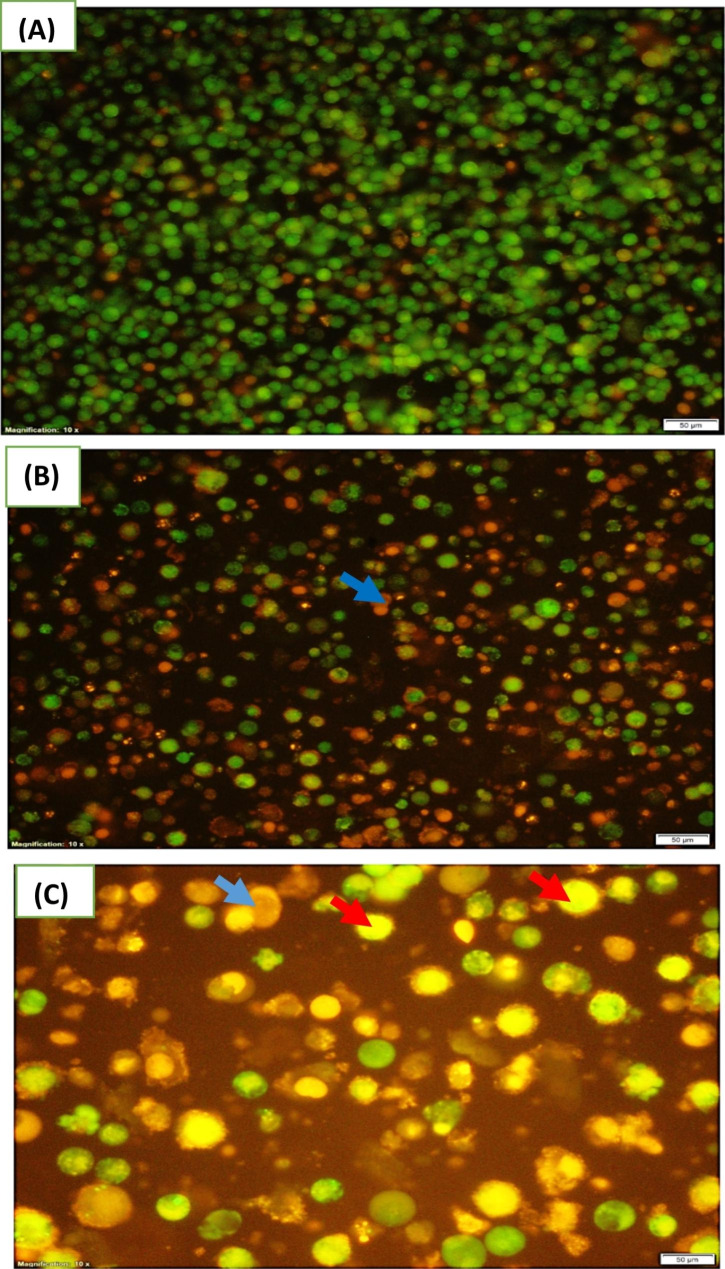
CaCo2 cells incubated in the absence (**A**) showing green nuclei, or the presence of the IC50 of LA-EPS-20079 (**B**). The later treatment induced apoptosis and some cells showed early apoptosis (red arrows) and others presented late apoptosis (blue arrow) (**C**). Images were examined via a fluorescence microscope

During apoptosis, nuclear changes were also observed and photographed using a fluorescence microscope following the application of AO/EB protocol. In the normal living cells, nuclei stained green with also green chromatin and they presented organized structures, however, in the early apoptotic phase, cells displayed fragmented or even condensed chromatin stained either green or orange. Nevertheless, during either the late apoptotic or even the necrotic stages, cells presented similar normal nuclei staining like live cells but with orange chromatin. Upon treating the CaCo-2 cells with the IC50, they showed an early apoptotic feature in the form of orange-stained multinucleated cells (Fig. 9). Comparable results were reported by Mathi et al. [32].

### Effect of ***Lactobacillus*** oligosaccharide on gene expression level

**Fig. 10:**
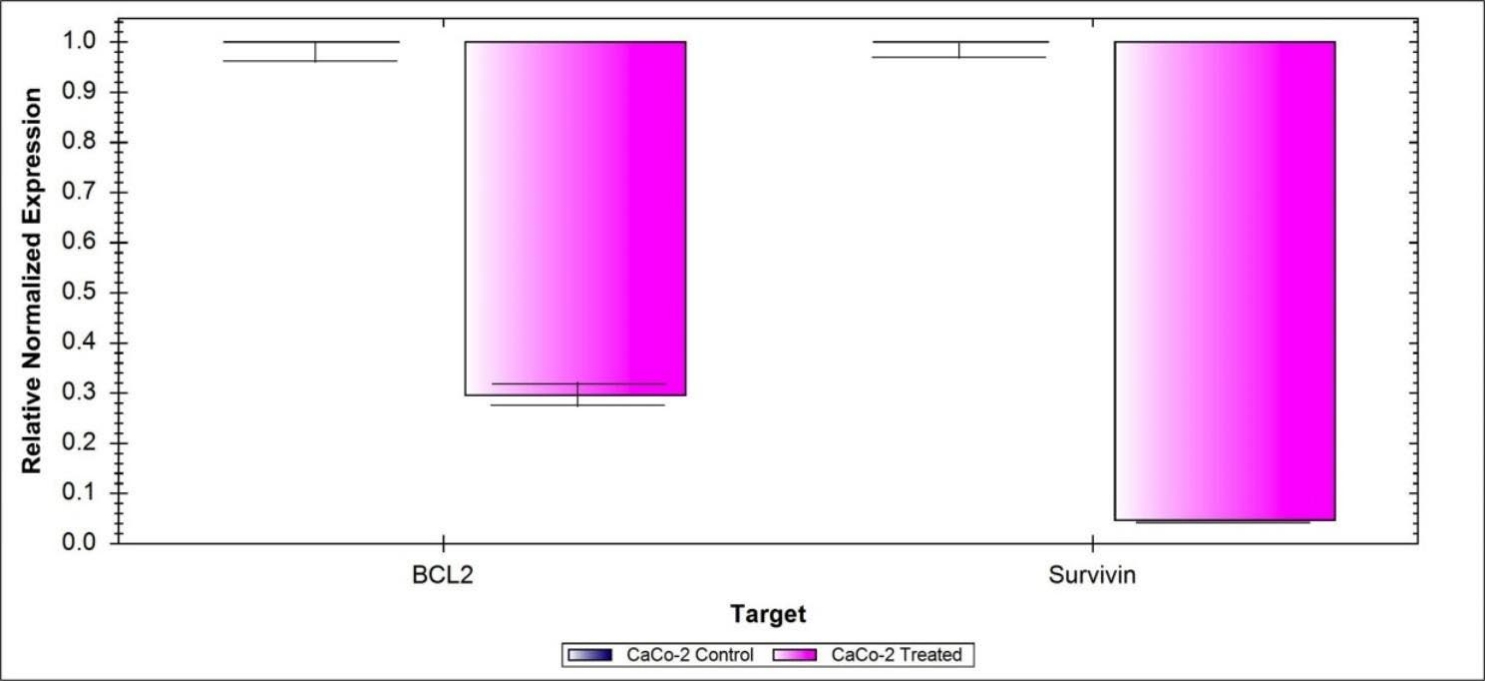
Relative gene expression RT-qPCR results for *BCL2* and *Survivin* genes in oligosaccharide-treated CaCo-2 cell lines relative to the untreated control CaCo-2 cells. The gene expression data was normalized using the *GAPDH* reference gene

In order to evaluate the effect of *Lactobacillus* oligosaccharides on the relative gene expression levels of *BCL*_*2*_ and *Survivin* genes, RT-qPCR was performed. The results presented in Fig. 10 indicate that both genes were down-regulated in the treated cell lines relative to the untreated control cells with down-regulation fold change of 3.377 and 21.38 for both *BCL*_*2*_ and *Survivin* genes, respectively in the treated cells relative to the control cells. This result indicated that *Lactobacillus* oligosaccharide treatment showed induce cellular apoptosis in human colo-rectaladeno carcinoma cell line (CaCo-2) via down-regulating the expression of both *Survivin* and *BCL*_*2*_ genes in the cancer cell line. Cell death intrinsic pathway is closely linked to the *BCL*_*2*_ anti-apoptotic activity. A research report the transport of *Bax* (pro-apoptotic) protein into the mitochondrial membrane that resulted in depolarization and cytochrome C release, as a result of this translocation, cell death steps were induced [33]. *Survivin* and *BCL*_*2*_ hold potent anti-apoptotic characters. It was mentioned that Survivin affects caspase-3 or − 7 through the expression of *BCL*_*2*_ family. In addition, in epithelial ovarian cancer, a positive correlation between the expression of both *BCL*_*2*_ and *Survivin* genes was found [33]. So, our results indicated that apoptosis related gene *BCL*_*2*_ may have a synergic role with *Survivin* gene in controlling colon cancer after oligosaccharide treatment.

## Conclusion

In the current study, the composition of the MRS media was optimized using Plackett Burman and Box–Behnken designs to maximize the production of LA-EPS. Glucose, yeast extract and sodium acetate trihydrate were the most significant variables that affecting production of LA-EPS compared to the other variables. Maximum production of LA-EPS-20079 oligosaccharides by *L*. *acidophilus* was achieved on production medium that consists of in g/l: 30, glucose; 1.0, peptone; 10.0, beef extract; 5.0, yeast extract; 0.2, K_2_HPO_4_; 0.2, triammonium citrate; 10.0, sodium acetate trihydrate and 2.0, MgSO_4_.7H_2_O. Additionally, the optimum physical variables were temperature, 30 °C; pH, 6 and incubation time, 48 h. The highest concentration of LA-EPS reached 794.82 μg/ml on optimized production medium. The previous reported anticancer activity of LA-EPS against colon cancer cell line (by our research team) is confirmed after performing the optimization process.

## Data Availability

The raw data supporting the conclusions of this article will be made available by the authors, without undue reservation, to any qualified researcher.
